# Supplementary material for: Dietary Patterns Associated with Abnormal Glucose Tolerance following Gestational Diabetes Mellitus: The MyNutritype Study
Source: Nutrients. 2023 Jun 20;15(12):2819. doi: 10.3390/nu15122819 (PMC10302866; doi:10.3390/nu15122819)
Supplement: Supplementary file 1 [file nutrients-15-02819-s001.zip › nutrients-2428282-supplementary.pdf]

Table S1. Factor loading scores for dietary patterns identified by principal component analysis

Food Groups	Dietary Patterns and Factor Loading Scores				
	**DP 1 (Unhealthy)**	**DP 2 (Fish-Eggs-Fruits-Vegetables)**	**DP 3 (Cereals-Confectionaries)**	**DP 4 (Legumes-dairy)**	**DP 5 (Meat-Sugar-Sweetened Beverages)**
Cereals and grains	0.192	0.049	**0.705**	0.088	0.293
Fast food	**0.628**	0.017	0.243	0.176	0.158
Meat and poultry	−0.104	0.201	−0.089	−0.176	**0.774**
Fish and seafood	0.006	**0.687**	−0.004	−0.025	−0.111
Eggs	0.096	**0.643**	−0.026	−0.047	0.244
Legumes	−0.050	0.019	0.072	**0.872**	−0.081
Milk and dairy products	**0.325**	0.293	**−0.474**	**0.463**	0.122
Fruits and vegetables	−0.012	**0.648**	0.283	0.230	0.040
Coffee, tea, and sugar-sweetened beverages	**0.313**	−0.174	0.236	0.265	**0.535**
Confectionaries	0.209	0.207	**0.627**	0.012	−0.246
Sugar, honey, bread spreads, and creamer	**0.819**	−0.133	0.020	−0.051	−0.011
Condiments	**0.720**	0.285	0.097	−0.072	0.029

DP: dietary pattern. Bold indicates factor loading score ≥0.3 (high intake of dietary pattern) or ≤-0.3 (low intake of dietary pattern).
